# Classification of Microglial Morphological Phenotypes Using Machine Learning

**DOI:** 10.3389/fncel.2021.701673

**Published:** 2021-06-29

**Authors:** Judith Leyh, Sabine Paeschke, Bianca Mages, Dominik Michalski, Marcin Nowicki, Ingo Bechmann, Karsten Winter

**Affiliations:** ^1^Institute of Anatomy, University of Leipzig, Leipzig, Germany; ^2^Department of Neurology, University of Leipzig, Leipzig, Germany

**Keywords:** microglia, morphology, machine learning, stroke, hippocampus, cortex

## Abstract

Microglia are the brain’s immunocompetent macrophages with a unique feature that allows surveillance of the surrounding microenvironment and subsequent reactions to tissue damage, infection, or homeostatic perturbations. Thereby, microglia’s striking morphological plasticity is one of their prominent characteristics and the categorization of microglial cell function based on morphology is well established. Frequently, automated classification of microglial morphological phenotypes is performed by using quantitative parameters. As this process is typically limited to a few and especially manually chosen criteria, a relevant selection bias may compromise the resulting classifications. In our study, we describe a novel microglial classification method by morphological evaluation using a convolutional neuronal network on the basis of manually selected cells in addition to classical morphological parameters. We focused on four microglial morphologies, ramified, rod-like, activated and amoeboid microglia within the murine hippocampus and cortex. The developed method for the classification was confirmed in a mouse model of ischemic stroke which is already known to result in microglial activation within affected brain regions. In conclusion, our classification of microglial morphological phenotypes using machine learning can serve as a time-saving and objective method for post-mortem characterization of microglial changes in healthy and disease mouse models, and might also represent a useful tool for human brain autopsy samples.

## Introduction

Microglia serve as the central nervous system (CNS)’s immunocompetent macrophages, which crucially contribute to homeostasis, plasticity, and learning by taking up pathogens, apoptotic cells, synaptic remnants, toxins, and myelin debris (Bradl and Lassmann, 2010; Sofroniew and Vinters, 2010; Goldmann and Prinz, 2013; Parkhurst et al., 2013; Nutma et al., 2020; Traiffort, 2020). Our current understanding is that these highly specialized brain-resident immune cells constantly monitor the brain’s microenvironment enabling them to detect and respond to tissue damage, infection, or homeostatic perturbations (Nimmerjahn et al., 2005). In the scanning state and under physiological conditions, microglial morphology is characterized by a small cell body and very fine, highly ramified processes, which allow these cells to screen their local brain parenchyma for signs of pathogens or cellular damage. In this steady-state condition, highly branched microglia were previously described as “resting”, but recent studies revealed them to be greatly dynamic and microglia should rather be described as “surveilling” cells (Tremblay et al., 2011; Nimmerjahn, 2012). So-called damage-associated molecular patterns (DAMPs), which are warning mechanisms in the form of secreted or released molecules from pathogens and injured cells, initiate microglial immune responses triggering process retraction, cell soma size increase and thickening, and morphological transformation from a ramified toward an activated morphology and finally to an amoeboid cell form (Huang et al., 2015; Colonna and Butovsky, 2017). Amoeboid microglia are characterized by completely retracted processes and a swollen cell soma (Doorn et al., 2014). The rapid morphological transformation of microglia enables these cells to migrate to the site of injury or to phagocytose harmful debris and invaders (Davalos et al., 2005; Nimmerjahn et al., 2005; Tremblay et al., 2011). Remarkably, between the two classes at the ends of the microglial morphology spectrum, of either ramified or amoeboid cell shape, microglia exhibit a variety of morphological transition states, which may reflect disease-specific functional cell states, but their spatial organization and precise role in the damaged or diseased brain is still unclear (Stence et al., 2001; Fumagalli et al., 2013; Salamanca et al., 2019). Recent studies described a fourth morphology of microglia in mice, so-called rod-like microglial cells, which were already reported by Franz Nissl in 1899 (Nissl, 1899; Ziebell et al., 2012; Rojas et al., 2014; Bachstetter et al., 2017; Holloway et al., 2019). Rod-like microglia do not exhibit planar processes and show a decreased number of secondary branches as well as narrowing of cell and soma (Ziebell et al., 2012; Taylor et al., 2014).

Microglial cells are active participants in various pathological conditions such as neurodegenerative disorders, traumatic brain injury, and stroke. Ischemic stroke due to obstruction of blood vessels is a leading cause of morbidity and mortality worldwide and not only affects neurons, but also the glial network including microglia (del Zoppo, 2009; Deb et al., 2010; Campbell et al., 2019). Along with cerebral ischemia a rapid deramification of microglial cells occurs, while severe ischemic stroke is accompanied by an intense microgliosis followed by the production of both neuroprotective and detrimental mediators (Masuda et al., 2011; Zhao et al., 2017; Zhang, 2019). Activated microglia may be involved in the progression of the ischemic lesion, but their precise function during ischemia evolution remains unclear. While single-cell RNA sequencing recently highlighted the whole range of microglial functions reflected by their phenotypic diversity and comprehensively characterized these cells at the molecular level, it does not provide the spatial information for a full understanding of brain homeostasis and disease progression mechanisms. Physiological and pathological conditions including regional distribution, species specificity, neurological disorders, and CNS tissue injuries can affect microglial heterogeneity (Grabert et al., 2016; Galatro et al., 2017; Gosselin et al., 2017; Soreq et al., 2017; Sousa et al., 2017; Heindl et al., 2018; Masuda et al., 2020).

There are numerous studies on automated detection and quantification of Iba1 or CD11b-positive cells in healthy or injured brain in rodents (Kozlowski and Weimer, 2012; Valous et al., 2013; Kongsui et al., 2014; Rey-Villamizar et al., 2014; Johnson and Walker, 2015; Zanier et al., 2015; Ding et al., 2017; Morrison et al., 2017; York et al., 2018; Kyriazis, 2019). Automated classification of microglial morphological phenotypes is performed by using quantitative parameters like *convex hull area*, *soma perimeter*, *process length*, *number of processes*, *process branching process volume*, *circularity, solidity, fractal dimension* and, *lacunarity* (Kongsui et al., 2014; Zanier et al., 2015; Fernández-Arjona et al., 2017, 2019; Morrison et al., 2017; York et al., 2018; Kyriazis, 2019). However, as these approaches hold the risk for a selection bias due to the naturally limited number of criteria and their manual selection during a single experiment, more elaborated concepts are needed to achieve the best possible accuracy in morphological classifications.

We here describe a novel classification method for analysis of microglial phenotypes by morphological evaluation using machine learning within the murine hippocampus and cortex with a focus on four microglial morphologies (ramified, rod-like, activated, amoeboid). In addition to classical morphological parameters, we used a convolutional neuronal network (CNN) for the classification of microglial phenotypes on the basis of manually selected cells. CNNs were already used for phenotype classification, for example for images of intracellular actin networks (Oei et al., 2019), multichannel single-cell images (Dürr and Sick, 2016), and Iba1-immunopositive microglia (Kyriazis, 2019). To confirm a reliable classification of different microglial morphological phenotypes, we finally applied our developed method in a mouse model of ischemic stroke which is already known to result in microglial activation.

## Materials and Methods

### Animals and Diets

The experiments were performed using male wild-type C57BL/6J mice (*n* = 36) and leptin receptor-deficient *db/db* (*n* = 37) and Lepr^*db*^/+ (*db/+*) (*n* = 36) mice as well as male wild-type C57BL/6J mice which underwent 24 h of transient focal cerebral ischemia (*n* = 6) by occlusion of the middle cerebral artery (MCA) as described in Mages et al. (2021). In our study, the filament occluding the MCA was retracted after 1 h of ischemia, and reperfusion was allowed until animals were sacrificed 24 h after ischemia induction. All animals were kept in the local animal facility under standard conditions: 12 h dark/light cycle, group-housed with free access to water and food. We performed this study in accordance with the guidelines of the Animal Experimental Committee following the German Animal Welfare Act as well as the European guidelines (Directive 2010/63/EU) concerning the protection of laboratory animals. The study was carried out in compliance with the ARRIVE guidelines. All experimental procedures and protocols were authorized by the local ethics committee of the state of Saxony (Landesdirektion Sachsen, Leipzig, approval nos. TVV 65/15, TVV 02/17, and TVV 41/17).

### Tissue Preparation

Mice were anesthetized with isoflurane (Baxter GmbH, Unterschleißheim, Germany) and transcardially perfused with ice-cold phosphate buffered saline (PBS, pH 7.4) and 4% paraformaldehyde (PFA) in 0.2 M PBS. Brains were carefully removed from the skull and post-fixed for 24 h in 4% PFA in 0.2 M PBS. Perfused and fixed brains of male wild-type C57BL/6J mice as well as *db/db* and *db/+* mice were sliced into 20 μm thick coronal or horizontal floating sections using a vibratome (Leica VT 1200, Leica Biosystems, Wetzlar, Germany) before their storage in PBS, containing 0.2% sodium azide, until further processing. Tissue preparation of C57BL/6J mice subjected to experimental cerebral ischemia was performed as described in Mages et al. (2021).

### Staining

For staining with rabbit anti-Iba1 (Synaptic Systems, Göttingen, Germany) to label microglia, floating brain sections were mounted onto microscopic slides followed by three wash steps with 0.3% Triton X-100 in 0.02M PBS for 10 min each time. Then, slices were incubated for 20 min in PBS containing 1.5% hydrogen peroxide at room temperature in order to quench the endogenous peroxidase activity. Afterward, brain sections were washed again three times with 0.3% Triton X-100 in PBS for 10 min each time, and slices were subsequently pretreated with 0.5% sodium borohydride in PBS for 30 min to reduce background staining. Thereafter, slices were thoroughly rinsed in 0.3% Triton X-100 in PBS and were blocked for 1 h in PBS blocking buffer containing 5% normal goat serum and 0.3% Triton X-100 at room temperature. Then, brain sections were incubated with the primary antibody Iba1 (1:500) diluted in PBS with 5% of normal goat serum. Incubation was done overnight at 4°C. The next day, brain sections were rinsed three times with 0.3% Triton X-100 in PBS and incubated with the biotinylated goat anti-rabbit IgG secondary antibody (1:100; Vector Laboratories, Burlingame, CA, USA) for 1 h at room temperature. After three wash steps with 0.3% Triton X-100 in PBS for 10 min each time, slices were incubated with VECTASTAIN Elite ABC HRP Kit (Vector Laboratories, Burlingame, CA, USA) for 30 min at room temperature. Thereafter, sections were washed with PBS and 0.05 M Tris, stained 5 min with the Vector SG HRP substrate (Vector Laboratories, Burlingame, CA, USA) producing a blue-gray reaction product, and were thoroughly rinsed in Tris and distilled water. Finally, brain sections were dried and covered with Entellan (Toluene; Merck KGaA, Darmstadt, Germany) and coverslips. For negative controls, the omission of primary antibodies, under otherwise identical conditions, resulted in the absence of any labeling (data not shown). A critical step for successful cell detection and classification was the reduction of background staining, which we overcame by performing a pre-treatment with 0.5% sodium borohydride in PBS. Prior to this, other microglia-specific markers (P2RY12, TMEM119) were also explored but did not result in desired image quality regarding the resolution of cell processes and subsequent cell detection.

To define the ischemic area, which was subsequently used for microglial classification, the proteins MAP2 (microtubule-associated protein 2), NF-L (neurofilaments-light chain), and collagen IV (Coll IV) were used as ischemia-sensitive markers (Popp et al., 2009; Härtig et al., 2016, 2017; Mages et al., 2018) in animals which underwent transient MCA occlusion. In general, fluorescence staining was performed as described in Mages et al. (2018), whereas following antibodies and dilutions were used (Mages et al., 2018). Primary antibodies: Rabbit-anti-neurofilament L (1:200, Synaptic Systems, Göttingen, Germany); mouse-anti-MAP2 (clone HM-2; 1:500, Sigma, Taufkirchen, Germany); rabbit-anti-collagen IV (1:100, Merck Millipore, MD, USA). Secondary antibodies: AlexaFluor488-donkey-anti-mouse IgG, AlexaFluor586-donkey-anti-rabbit IgG, AlexaFluor647-donkey-anti-goat IgG, each 1:250, each Thermo Fisher, Waltham, MA, USA. Brain sections were scanned with an Axio Scan.Z1 slide scanner (Carl Zeiss Microscopy GmbH, Jena, Germany) and files were exported using the NetScope Viewer Pro Software (Net-Base Software GmbH, Freiburg i. Br., Germany). In line with earlier reports (Härtig et al., 2017; Mages et al., 2018) the ischemic area was characterized by a loss of MAP2 (Supplementary Figure 1A), whereas the NF-L- and Coll IV-related immunofluorescence intensities increased in these regions compared to the non-ischemic contralateral hemisphere (Supplementary Figures 1B,C). Supplementary Figure 1D shows the selected neocortical and hippocampal areas within the ipsilateral and contralateral hemispheres.

### Image Acquisition and Processing

**Figure 1:**
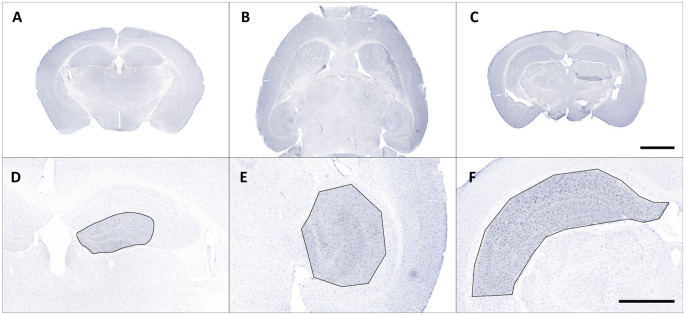
Representative overview photomicrographs showing Iba1 staining in coronal **(A,C)** and horizontal **(B)** brain sections of male C57BL/6J mice **(A)** *db/+* mice **(B)** and C57BL/6J mice with ischemia **(C)**. Exemplary regions of interest (ROIs) were manually selected in corresponding images **(D–F)**. Scale bars represent 2,000 μm **(A–C)** and 1,000 μm **(D,E)**.

Iba1-stained brain sections were fully digitalized using a digital slide scanner (Pannoramic Scan II, 3D HISTECH Ltd., Budapest, Hungary) at 40× magnification and automatically stitched (Figures 1A–C). The scanner software (Pannoramic Scanner, version 1.23, 3D HISTECH Ltd., Budapest, Hungary) was operated in extended focus mode (eight levels with 1 μm axial distance) to combine images from several adjacent focal planes into one image with maximum depth of sharpness. This procedure enables coherent imaging of freely aligned cell processes within a shallow tissue volume instead of producing images with interrupted processes from a single focal plane.

Regions of interest (ROIs) were manually selected and corresponding images were exported from slide scanner data sets (Case Viewer, version 2.3, 3D HISTECH Ltd., Budapest, Hungary) with a pixel dimension of 0.122 μm (Figures 1D–F). Exported images were converted to grayscale and submitted to contrast limited adaptive histogram equalization (CLAHE; Heckbert, 1994) using Icy (version 2.0.3^1^, de Chaumont et al., 2012). The resulting images were imported in Mathematica (version 11.2, Wolfram Research, Inc., Champaign, IL, USA), grayscale colors were inverted and tissue area was computed. Soma detection was performed in two steps. First, a series of top hat (Gonzalez and Woods, 2016) and Gaussian filter operations was applied to the inverted images to suppress cell processes and enhance cell somata. Processed grayscale images were then binarized using Otsu’s (cluster variance maximization) thresholding method (Otsu, 1979). The binarized images were cleared of smaller segments that did not match somata (artifacts or clumped cell processes) by using an empirically determined size threshold of 1,500 pixels and the remaining somata were reconstructed by morphological closing (Gonzalez and Woods, 2016) within a 7.5-pixel radius.

Process detection was performed in a hybrid fashion to preserve connections between cell somata and cell processes. In the first step, the inverted images were submitted to local adaptive segmentation (5-pixel radius) to detect all stained cells. In the second step, all processes within the inverted images were amplified by using a ridge-detecting image filter (“RidgeFilter”, *σ* = 5) to enhance local structural coherence and the resulting images were also submitted to local adaptive segmentation (5-pixel radius). Both segmented images were subsequently added and merged with the respective somata image. Since some processes may appear separated from somata due to the imaging procedure, an additional reconstruction step was performed by connecting endpoints of processes to the respective somata within a 50-pixel radius. In the last step all images were cleared of processes without connections to any somata and all cells intersected by the border of the image area were removed.

The resulting images contained many connected cells which had to be separated from each other. Centroid coordinates of all somata were calculated and used as seed pixels for a parallel flood fill operation. Starting from the seeds this operation fills all pixels of the detected cells with a unique label, either to the cell borders or to the filling fronts of connected cells. After this step, all individual cells of an image were uniquely labeled. All final cell segmentations along with results of relevant intermediate steps were examined to ensure proper processing and detection.

**Figure 2:**
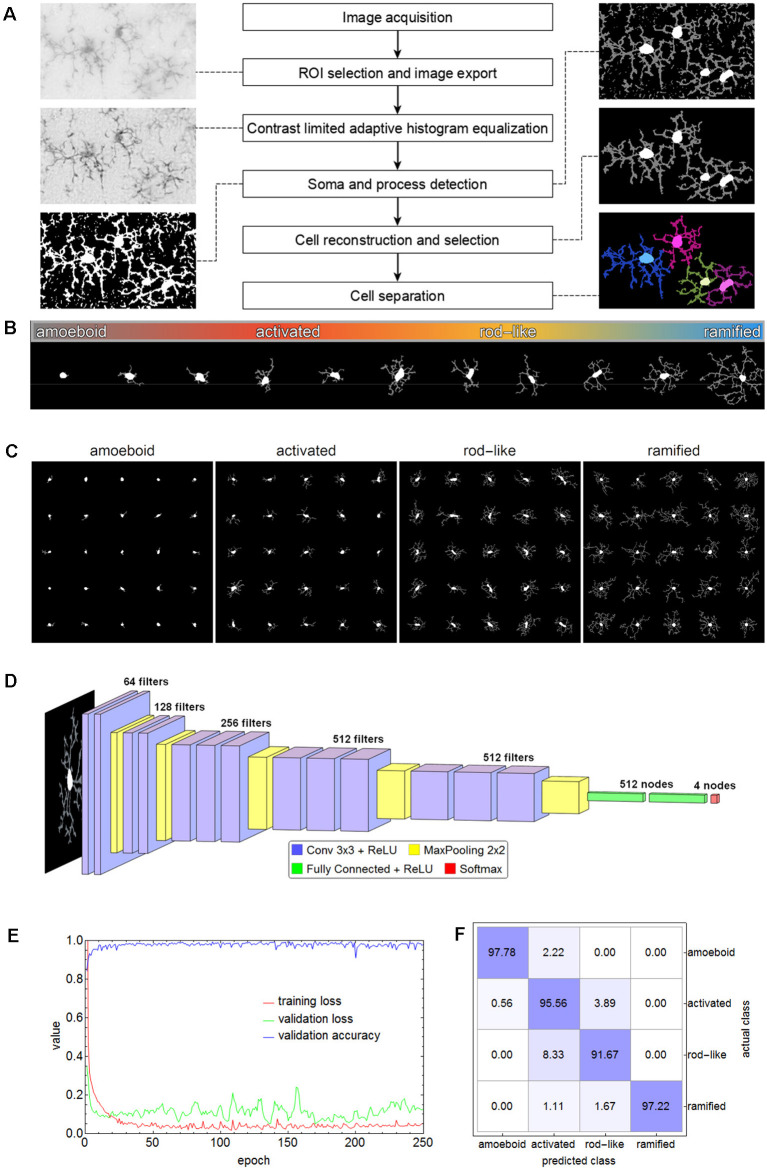
Flow chart of all steps involving the segmentation of microglial cells **(A)**. The spectrum of the phenotypic diversity of microglial cells after segmentation **(B)**. Random samples from cells that were manually selected as the basis for cell classification **(C)**. The convolutional neural network (CNN) based on the VGG-16 architecture that was used for cell classification **(D)**, along with the performance parameters (log-loss and accuracy) during the training **(E)** and the confusion matrix for the test set **(F)** with an overall accuracy of 95.56%.

For subsequent cell classification, the cells of all images were cropped from the original image area and exported as binary masks containing their complete shape (as well as in separate shapes for soma and processes, respectively) along with their original grayscale representation from the equalized grayscale images. Additionally, for visual inspection cell shapes were submitted to morphological thinning to compute the medial axes, the so-called skeleton, of all processes which was combined with the respective somata. Figure 2A shows a schematic overview of the applied methods.

### Cell Classification of Microglial Cells in Wild-Type C57BL/6J, *db/db*, and *db/+* Mice

Microglial cells express a considerable phenotypic diversity (Figure 2B). After thorough inspection of all exported cell images, 1,000 cells per class were manually selected as the basis for cell classification and the corresponding cell images were cropped and rescaled to 128 × 128 pixels (Figure 2C). Images were split into training (70%), validation (15%), and test (15%) set. The test set was only used for the evaluation of the trained network. Images belonging to the training set were submitted to image augmentation (Shorten and Khoshgoftaar, 2019) to expand data diversity and make the classification more robust (Gao et al., 2017). A series of rotation and reflection image transforms was applied to each image, and after image augmentation, 3-fold cross-validation was performed.

A convolutional neural network (CNN) based on the VGG-16 architecture was selected for cell classification (Simonyan and Zisserman, 2015). The network consisted of 13 convolutional layers, five max-pooling layers, two fully connected layers, and a softmax layer of four nodes for the classes amoeboid, activated, rod-like, and ramified (Figure 2D). ReLU was used as an activation function and after each activation, BatchNormalization was applied for regularization. “Adam” optimizer was used for optimization, the initial learning rate was set to 0.001, batch size was set to 64, and a dropout rate of 0.5 was applied to constrain the fully connected layers and to reduce overfitting. The CNN was trained on an off-the-shelf NVIDIA GeForce GTX 1080 with 8 GB GPU memory for 250 epochs, training time took about 5.8 h. The performance parameters (log-loss and accuracy) are shown in Figure 2E. Averaged values of the last 50 training rounds were as follows: training loss 0.0497, validation loss 0.2887, and validation accuracy 0.9726. Subsequently, the test set was submitted to the trained CNN. Overall accuracy was 95.56%, the confusion matrix is shown in Figure 2F. While 97.78% of the amoeboid and 97.22% of the ramified cells were correctly classified, the percentage dropped to 95.56% and 91.67% for activated and rod-like cells, respectively. The matrix shows that 3.89% of activated cells were misclassified as rod-like cells, while 8.33% were misclassified vice-versa, indicating the more prominent phenotype overlap between these two classes.

### Classification and Quantitative Analysis of Microglial Cells in Ischemia Affected Regions

**Figure 3:**
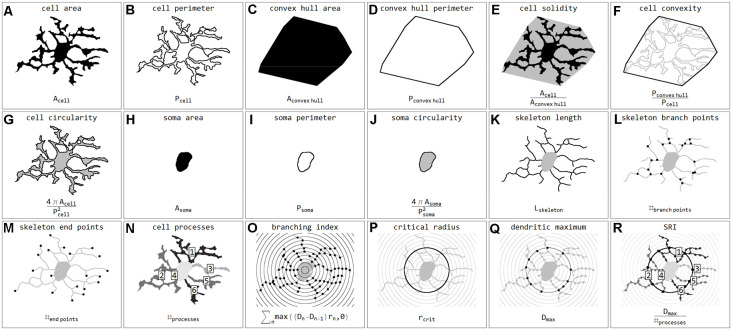
Illustrations and formulas of all morphological parameters used for quantification of microglial cells (inspired by Fernández-Arjona et al., 2017). Parameters based on area **(A,C,H)**, perimeter **(B,D,I)**, combinations of area and perimeter **(E,F,G,J)**, skeleton **(K,L,M)**, processes **(N)**, and Sholl analysis **(O,P,Q,R)**.

Stroke sections were submitted to the same image acquisition and cell extraction procedure mentioned above. Brain sections and ROIs were selected based on the ischemia-induced decrease of MAP2-related and increase of NFL- and Coll IV-related immunofluorescence intensities within cortical and hippocampal regions (Supplementary Figure 1). These regions were mirrored to the contralateral control hemisphere, thus capturing four ROIs per animal (Mages et al., 2021). The selection was performed and verified by experienced investigators. Exported cell masks (soma white, processes gray) were also scaled to 128 × 128 pixels and submitted to classification. In total 15,786 single cells from 24 stroke ROIs were classified. Individual cells were coded as labeled regions within the original image area. Labels were subsequently color-coded according to classification results. Final images were used for visualization and classification verification. Subsequently, cells were submitted to quantification and all calculated parameters are presented in Figure 3. Parameters include *areas* (μm^2^) and *perimeters* (μm) of whole cells (Figures 3A,B), their *convex hulls* (the smallest convex set of pixels that encloses a cell; Figures 3C,D) and their somata (Figures 3H,I); *cell solidity* (the degree to which the *area* of a cell fills the area of its *convex hull*; Figure 3E) and *convexity* (the ratio of a cell’s *convex hull perimeter* to the cell’s actual perimeter; Figure 3F); *circularity* of cells and somata (the roundness, where 1 equals a perfect circle and values smaller than 1 indicate shapes that increasingly deviate from the shape of a circle; Figures 3G,J); *length* (μm) as well as the number of *branch* and *endpoints* (*n*) of the skeletonized *processes* (Figures 3K–M); and the number of *cell processes* (*n*; Figure 3N). The number of cell processes was calculated by subtracting the dilated soma (3-pixel dilation) from the respective cell and counting the number of all isolated processes. Furthermore, all cells were submitted to Sholl analysis (Sholl, 1953) and the cell’s* branching index* (Garcia-Segura and Perez-Marquez, 2014; a measure to distinguish between cells of different ramification types; Figure 3O), *critical radius* (μm; the radius with the maximum number of process crossings; Figure 3P), *dendritic maximum* (*n*; the number of process crossings at the critical radius; Figure 3Q) and the *Schoenen ramification index* (*SRI*, Schoenen, 1982; a measure of the branching of a cell; Figure 3R) were calculated. Additionally, for whole images the segmented image area (%), representing the ratio of segmented pixels within the total image area (before cell detection), and the cell density (cells per mm^2^) were computed.

### NC (Nearest Centroid) Classification

**Figure 4:**
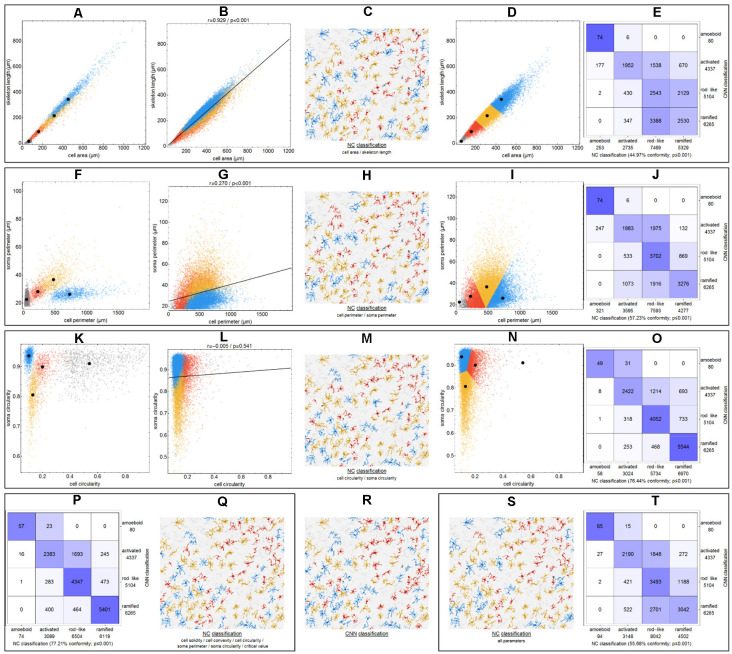
Differences between CNN classification and NC (nearest centroid) classification for various parameter combinations. Scatter plots with respective class centroids (black points) of all manually selected cells **(A,F,K)**. Scatter plots of all cells after CNN classification **(B,G,L)** with regression line. Scatter plots of all cells with initial class centroids (black points) after NC classification **(D,I,N)**. Comparison of CNN and NC classification results with degree of conformity and p-value from exact symmetry test for paired contingency tables **(E,J,O,P,T)**. Example image section with color-codings for CNN classification **(R)** and NC classifications **(C,H,M,Q,S)**. Ramified microglia are depicted in blue, rod-like cells in orange, activated microglial cells in red, and amoeboid cells in gray.

To demonstrate the differences between CNN classification and conventional parameter-based classification, we applied an NC classification method to various parameter combinations (Figure 4). Morphological parameters of all manually selected cells that were used for the training of the CNN were calculated.

**Figure 5:**
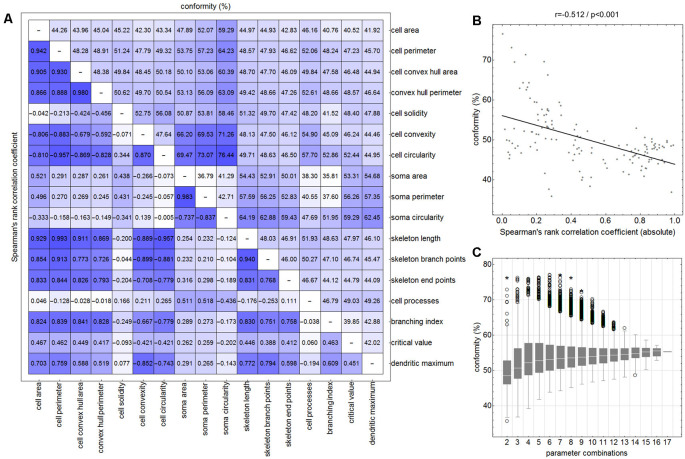
Degree of conformity (CNN vs. NC classification; upper triangular matrix) and correlation coefficients (correlation for the distribution of all cell’s parameter points within the parameter space; lower triangular matrix) of all two-parameter combinations **(A)**. Linear regression analysis between the degree of conformity (CNN vs. NC classification) and the absolute correlation coefficient (distribution of all cell’s parameter points within the parameter space) for all two-parameter combinations **(B)**. Ranges of conformity values for 2–17 parameter combinations **(C)**. Circles represent outliers and asterisks represent extreme values.

Initially—and to demonstrate the approach—we considered combinations consisting of only two parameters. Figure 4 shows results for the following combinations: *cell area/skeleton length* (A–E), *cell perimeter/soma perimeter* (F–J), *cell circularity/soma circularity* (K–O). In the first step, scatter plots for parameter values of all manually selected cells were generated using a color scheme for the indication of the four classes (Figures 4A,F,K; ramified microglia: blue, rod-like microglia: orange, activated microglia: red, amoeboid microglia: gray). Class centroids (median values of the current parameters) were calculated for all four classes and added to the scatter plots (black dots). Subsequently, scatter plots were also generated for the same parameter combinations of all CNN classified cells (Figures 4B,G,L). NC classification was performed for all already CNN classified cells by calculating the distance of their parameter combinations to all four class centroids and assigning the class of the nearest centroid. The resulting NC clusters were also presented as scatter plots (Figures 4D,I,N) along with the four class centroids. Classification results were compared (Figures 4E,J,O) and the degree of conformity of both methods was determined by calculating the portion of consistently (sum of diagonal matrix values) to all (sum of all matrix values) classified cells. Additionally, the generated matrices were tested regarding their symmetry using the exact symmetry test for paired contingency tables (“nominalSymmetryTest”) from the “rcompanion” package for R. Classification differences between the two methods were also illustrated using an exemplary image section with the color coding from CNN classification (Figure 4R) and color codings from NC classifications (Figures 4C,H,M). Correlation analysis was performed for all two-parameter combinations using Spearman’s rank correlation coefficient to characterize the distribution of parameter points of all CNN classified cells. Scatter plots and respective regression lines of selected parameter combinations are shown in Supplementary Figure 3. The degree of conformity (upper triangular matrix) and correlation coefficients (lower triangular matrix) of all two-parameter combinations are shown in Figure 5A. Another correlation analysis was performed to investigate a potential association between the degree of conformity (CNN vs. NC classification) and the absolute correlation coefficient, or more precisely to answer the question whether NC classification accuracy can be correlated to the distribution of parameter points within the parameter space (Figure 5B).

Similar analyses (NC classification, degree of conformity, and test for matrix symmetry) were performed for combinations ranging from three up to 17 parameters (131,054 combinations in total; Figure 5C). The parameter SRI was omitted from all analyses since it could not be calculated for each cell (division by zero for cells with no processes). Furthermore, a number of parameter combinations, as well as singular parameters whose presence resulted in highest degrees of conformity, were identified.

### Statistical Analysis

Statistical analysis was performed with IBM SPSS Statistics (version 22, IBM Corp., Armonk, NY, USA) and R (version 3.6.1; R Core Team^2^). Images were separated into analysis groups. For each group, the number of cells per class were counted, relative class proportions were calculated and respective stacked bar charts were generated. Group comparisons of class percentages were performed using the non-parametric density equality test (Li et al., 2009; “ndpdenq”, 999 bootstrap replications) from the “np” package for R. Descriptive statistics were calculated and box plots were generated. Data were tested for normal distribution using the Shapiro-Wilk Test (segmented image area and cell density) and Kolmogorov-Smirnov Test (grouped parameter data), and group comparisons were performed using Kruskal Wallis and Mann-Whitney-U tests. To adjust the *p*-value for multiple comparisons, *post hoc* Bonferroni correction was performed. The number of analyzed animals is indicated as “*n*” in the figure legend. Data are presented as the median and interquartile range (IQR). Significance was set as follows: *p* < 0.05 *, *p* < 0.01 **, *p* < 0.001 ***, *p* < 0.0001 ****.

## Results

### Class Percentage of Microglial Morphological Phenotypes

**Figure 6:**
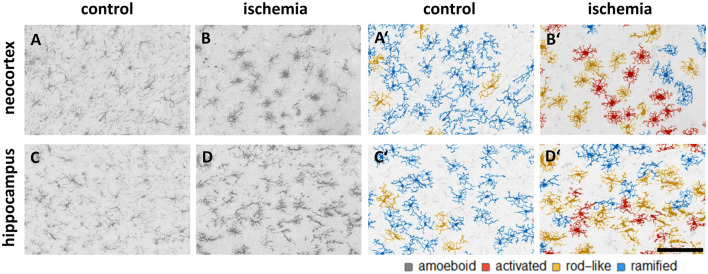
Representative images of Iba1 staining within the control neocortex **(A)**, ischemic neocortex **(B)**, control hippocampus **(C)**, and ischemic hippocampus **(D)** on the left side, and the appropriate microglial cell segmentation on the right side **(A′–D′)**. Ramified microglia are depicted in blue, rod-like cells in orange, and activated microglial cells in red. Amoeboid microglial cells are not present in the depicted sections due to their low occurrence (*n* = 80; see Supplementary Table 2).

**Figure 7:**
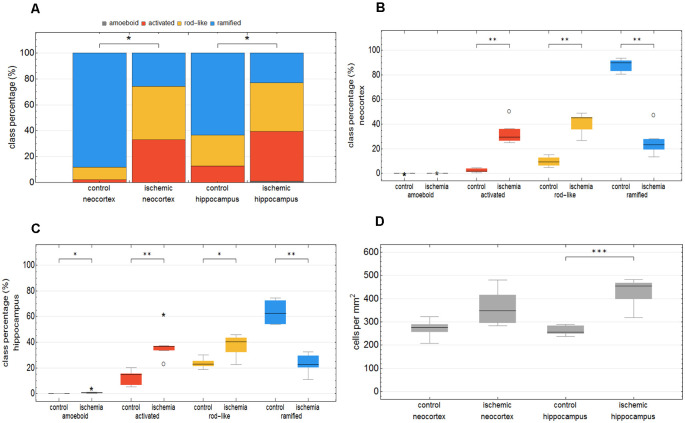
Total class percentage of microglial morphological phenotypes in control and ischemia-affected regions. Class percentage of ramified, rod-like, activated, and amoeboid microglia within the control neocortex, ischemic neocortex, control hippocampus, and ischemic hippocampus **(A)**. Individual class percentage of the four microglial morphological phenotype classes in the neocortex **(B)** and in the hippocampus **(C)**. Normalized microglial cell density in control and ischemia-affected regions **(D)**. *n* = 6 animals and about 16,000 analyzed cells. ***p < 0.001; **p < 0.01; *p < 0.05.

We trained the CNN with microglial cells of different mouse strains, male wild-type C57BL/6J mice, *db/db*, and *db/+* mice, to obtain and cover a wide variety of microglial morphological phenotypes. Subsequently, we examined our microglial classification method in a mouse model of experimental cerebral ischemia (24 h after ischemia induction) known for microglial activation in the area of ischemic tissue damage (Härtig et al., 2017; Zhang, 2019). Figure 6 shows representative images of Iba1 staining within the control neocortex (Figure 6A), ischemic neocortex (Figure 6B), control hippocampus (Figure 6C), and ischemic hippocampus (Figure 6D). Ischemia-affected regions in the hippocampus and neocortex presented more activated and rod-like Iba1-positive cells (which most probably correspond to microglia rather than infiltrated cells such as monocytes/macrophages at day one after experimental stroke; Jian et al., 2019; Rajan et al., 2019; Han et al., 2020) compared to the relevant brain areas within the contralateral hemisphere (Figures 6A′–D′). Classification of microglial morphological phenotypes using our neural network machine learning method confirmed the qualitative analysis of microglial phenotypes by morphological evaluation after staining with Iba1. Total class percentages within the ischemic neocortex and hippocampus were significantly different compared to the control neocortex or hippocampus (Figure 7A; control neocortex vs. ischemic neocortex *, control hippocampus vs. ischemic hippocampus *). Individual class percentages in the neocortex revealed significant increases of activated and rod-like microglial cells and a simultaneous decrease of ramified microglia within the ischemia-affected hemisphere compared to the control hemisphere. Amoeboid Iba1-positive cells did not differ between the ischemic and non-ischemic neocortex, as they were virtually absent (Figure 7B; activated microglia control vs. ischemic neocortex **, rod-like microglia control vs. ischemic neocortex **, ramified microglia control vs. ischemic **). Similarly, the percentages of activated and rod-like microglial cells within the ischemic hippocampus were significantly enhanced, whereas the amount of ramified microglia was lower compared to the control hippocampal area (Figure 7C; activated microglia control vs. ischemic hippocampus **, rod-like microglia control vs. ischemic hippocampus *, ramified microglia control vs. ischemic hippocampus **). In contrast to the neocortex, we also detected a significant increase in amoeboid microglial cells within the ischemic hippocampus compared to the control hippocampus (Figure 7C, amoeboid microglia control vs. ischemic hippocampus *). The segmented image area did not reveal any differences within the neocortical and hippocampal regions (Supplementary Figure 2). Normalized microglial cell density (cells per mm^2^) was significantly enhanced within the ischemic hippocampus compared to the control hippocampal area and a slight trend was observed in the neocortex (Figure 7D).

### Quantification of Morphological Parameters

Eighteen morphological parameters (*cell area, cell perimeter, convex hull area, convex hull perimeter, cell solidity, cell convexity, cell circularity, soma area, soma perimeter, soma circularity, skeleton length, skeleton branch poin*ts and *endpoints, cell processes, branching index, critical radius, dendritic maximum, SRI*) were measured for each detected Iba1-positive cell (*n* = 15786).

Firstly, we looked at all Iba1-positive cells within control and ischemic areas of the neocortex and hippocampus (Supplementary Table 1). Microglia within control and ischemic-affected hemispheres differed significantly regarding the morphological parameters. We found lower values for *cell area*, *perimeter*, *convex hull area*, *soma area*, *skeleton length*, *branch* and *endpoints*, *branching index*, *dendritic maximum* as well as *SRI* within the control and ischemic hippocampus compared to control and ischemic neocortex. Microglial *cell convexity* and *circularity* were greater in the hippocampus than in the neocortex. Control and ischemic areas in both brain regions showed differences in the morphology of microglial cells. Microglia had larger *cell perimeters*, *convex hull areas*, *soma circularities*, *skeleton lengths*, *skeleton branch* and *endpoints*, higher *branching indices*, and *SRI* within the control hemisphere compared to the ischemic-affected hemisphere (Supplementary Table 1).

To test whether our neural network machine learning method adequately classifies microglial cells into the four morphological phenotype groups, we merged all microglial cells of each morphological class within both control and ischemic brain regions. Supplementary Table 2 summarizes the 18 selected morphological parameters for amoeboid, activated, rod-like and ramified microglial cells. As expected, amoeboid microglia were characterized by the smallest values for *cell area*, *perimeter*, *convex hull area*, *soma area*, *skeleton length*, *branch* and *endpoints*, *branching index*, and *SRI* compared to the other morphological phenotypes. Amoeboid microglia’s *cell solidity* and *circularity* showed high values. The classified activated phenotype of microglia had a smaller *cell area*, *perimeter*, and *convex hull area* and also fewer *skeleton branches* and *endpoints* than ramified and rod-like microglial cells. Ramified microglia typically exhibit small somata and fine ramifications, which was demonstrated by a small *soma area*, big cell *convex hull area*, long *skeleton length* as well as a high *branching index*. Rod-like microglial cells projected similar *skeleton lengths*, *branch* and *endpoints* than ramified microglia, but exhibited a higher *cell* and *soma area* (Supplementary Table 2). All four morphological classes of microglia were significantly different among each other regarding the selected parameters.

**Figure 8:**
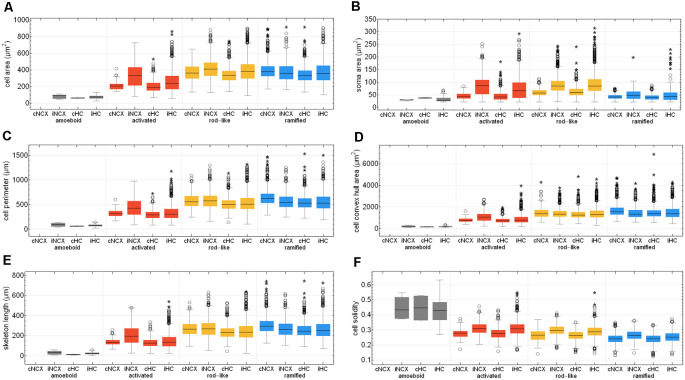
Quantitative analysis of six morphological parameters of classified microglial cells within control and ischemic neocortex and hippocampus. Cell area **(A)**, soma area **(B)**, cell perimeter **(C)**, cell convex hull area **(D)**, skeleton length **(E)**, and cell solidity **(F)**. cNCX, control neocortex; iNCX, ischemic neocortex; cHC, control hippocampus; iHC, ischemic hippocampus. Circles represent outliers and asterisks represent extreme values.

After Fernández-Arjona et al. (2019) had recently categorized activated microglial cells according to their morphometric parameters, we further looked at the activated morphotype in more detail (Fernández-Arjona et al., 2019). Ischemic-affected regions displayed more activated microglial cells, which showed smaller *cell* and *soma area*, *cell perimeter*, *convex hull area*, *skeleton length*, and *branching index* in the control area compared to the ischemic-affected corresponding area (Figures 8A–E, Supplementary Tables 3, 4). Activated microglial cells within the ischemic neocortex showed larger *cell* and *soma areas*, *cell perimeters* and *convex hull areas*, *skeleton lengths*, and higher *branching indices* compared to the ischemic hippocampus (Figures 8A–E, Supplementary Tables 3, 4).

*Cell area* of all microglial cells positively correlated with *soma area*, *cell perimeter*, *convex hull area*, *skeleton length*, but not with *cell solidity* (Supplementary Figures 3A–E). *Cell perimeter* positively correlated with *convex hull area*, *skeleton length*, and slightly negatively with *cell solidity* (Supplementary Figures 3F–H). *Convex hull area* positively correlated with *skeleton length* and is negatively associated with *cell solidity* (Supplementary Figures 3I,J). Control areas of both brain regions did not differ among each other concerning the above-mentioned six morphological parameters with the exception of *cell area*, *cell perimeter*, *convex hull area*, and *skeleton length* for ramified and rod-like cells (Figures 8A,C–E). Amoeboid microglia did not show any differences at all. We observed that rod-like microglia’s *cell* and *soma area* as wells as *cell solidity* are enhanced within the ischemic hemisphere compared to the control hemisphere (Figures 8A,B,F). In general, ramified cells exhibited small *soma areas*, big *cell perimeters*, and long *skeleton lengths* (Figures 8A,C,E).

Microglial *cell area* is expected to increase due to activation and soma enlargement yielding higher values of this morphological parameter for rod-like and activated microglia. The *cell perimeter* is estimated to be higher in ramified and rod-like cells. A decrease is characteristic of fewer ramifications. The more ramified is the microglial cell, the bigger is the *convex hull area*, thus leading to a smaller *cell solidity*. An increase of this parameter reveals the tendency of microglial cells to be more compact. *Cell circularity* is expected to be higher for amoeboid microglia. Typically, highly ramified microglia have a greater *skeleton length*, many *branches* and *endpoints*. The *branching index* is an additional measurement of microglial branching complexity. For instance, a small ramified microglial cell and an activated microglial cell may have a similar cell volume, but the activated microglia occupy more of its surrounding, therefore the *branching index* measure will be smaller. *SRI* constantly increases from an amoeboid toward an activated and rod-like morphology and to a ramified cell type (Figure 8 and Supplementary Table 2).

Since microglial cells are three-dimensional objects, the main limitation of our study is the two-dimensional way of image acquisition and subsequent image processing that does not properly allow to include all fine ramifications of different focal planes and can lead to underestimated cell parameters such as *area, perimeter*, or *skeleton length* for instance. Further, the thickness of brain slices is important to enable the analysis of entire microglial cells, each of which has its own territory of about 15–30 μm. Thin sections limit the accuracy of describing three-dimensional microglial morphology (Heindl et al., 2018). In line with Zanier et al. (2015), we used 20 μm and additionally 30 μm thick slices to ensure the detection of many no overlapping microglial cells (Zanier et al., 2015). Another just recently published study used even thinner brain sections of 7 μm thickness (Ding et al., 2017). We had problems to properly separate Iba1-positive cells from each other using thicker sections.

### Comparison of CNN and NC Classification

The comparison of CNN and NC classification revealed detailed information regarding the relationship between individual morphological parameters. Figure 4 and Supplementary Figure 3 show some selected scatter plots and Figure 5A (lower triangular matrix) provides correlation coefficients for all two-parameter combinations. Some parameters are closely related to each other (darker colored matrix cells; positively correlated: *cell perimeter* and *skeleton length*, negatively correlated: *cell perimeter* and *cell circularity*), while others show very weak correlation (lighter colored matrix cells; *cell area* and *cell solidity*). Parameters with close relationships and therefore strong absolute correlation coefficients express narrow spatial distributions near the regression line (Figure 4B). Parameters with weak to negligible absolute correlation coefficients tend to express broad and (but not always) less overlapping distributions (Figures 4G,L). NC classification is based on predefined centroids and cannot generate overlapping classes by design, which is a major pitfall for this approach. Therefore, NC classification results strongly depend on the shape and location of their respective point distributions in parameter space. This is also reflected in the comparison of CNN and NC classification in terms of their actual results: strongly correlating parameters show lower degrees of conformity (Figure 4E: 44.97%), while weakly correlating parameters show higher degrees of conformity (Figure 4O: 76.44%). Figure 5A (upper triangular matrix) provides values for the degree of conformity for all two-parameter combinations. As for correlation coefficients, these values are also emphasized with a color scheme and a certain pattern in relation to the matrix diagonal can be perceived. Correlation analysis of the degree of conformity (CNN vs. NC classification) and the absolute correlation coefficients revealed a moderate negative correlation (Figure 5B; *r* = −0.512, *p* ≤ 0.001). In addition to the numerical comparison of both approaches, color-coded cell images were also generated. Figure 4R shows a section with color coding according to CNN classification. Color codings after NC classification are also presented for the following combinations: *cell area* and *skeleton length* (Figure 4C), *cell perimeter* and *soma perimeter* (Figure 4H) as well as *cell circularity* and *soma circularity* (Figure 4M). Lower degrees of conformity result in greater deviations from the CNN color-coding and classification differences are distributed across all four classes with one recognizable accumulation: NC classification tends to classify a larger proportion of activated cells as rod-like cells (Figures 4E–O).

A clear limitation of the CNN classification approach presented in this study was the number of cells that were selected for the training of the neural network. Although image augmentation was performed to dramatically increase the number of images for the training and validation set, this procedure may not fully replace the addition of cells with a completely different morphology. To counteract subjective influences during the manual selection of training images, this procedure was performed by four experienced investigators resulting in a more diverse set of cells belonging to the four classes—although this approach might have introduced too much morphological variability and overlap. These two factors may have attributed to misclassification and might be addressed to increase overall classification accuracy.

All possible combinations with more than three and up to 17 parameters (131,054 combinations in total, SRI was omitted from all analyses) were also investigated (Figure 5C). Analyses revealed a peak degree of conformity at 77.21% with remaining NC overclassification of activated cells as rod-like cells (Figure 4P). However, the respective color-coding (Figure 4Q) largely resembles the CNN color-coding (Figure 4R). NC classification of all 17 parameters showed only a medium degree of conformity at 55.68% (Figure 4T), the respective color-coding is shown in Figure 4S. Certain parameters and combinations thereof are involved in NC classifications with higher—or even highest—degrees of conformity: *cell solidity, cell convexity, cell circularity, soma perimeter, soma circularity*, and *critical value*. Combinations of up to eight parameters could result in conformities of more than 75% (always with the participation of the parameters listed in the previous sentence), peak conformities of combinations with more parameters rapidly declined to less than 60%. The lowest conformities ranged down to 35.81% with parameters such as *skeleton branch points, skeleton endpoints* or *dendritic maximum*.

## Discussion

We can successfully confirm that our developed classification method of microglial morphological phenotypes works well by using a mouse model of transient MCA occlusion, which is one of the models that most closely simulate human ischemic stroke and is probably the most frequently used model in experimental stroke research (Engel et al., 2011; Fluri et al., 2015). Since microglial activation within the ischemia-affected brain regions has been well established (del Zoppo, 2009; Härtig et al., 2017; Zhang, 2019), this model was used as a positive control to confirm a reliable detection of activated microglia by using the established machine learning method. In our study, we did not analyze neurons, but we assumed that neuronal damage or neuronal death is likely in ischemia-affected regions where microglia show activation processes to engulf cellular debris. Here, we showed that ischemia-affected regions in the hippocampus and neocortex presented more activated and rod-like microglial cells and consequently less ramified microglia compared to the relevant brain areas within the contralateral hemisphere. Michalski et al. (2017) recently demonstrated that Iba1-staining density and intensity were strongly increased in the ischemic core and ischemic border zone compared to the control area located at the contralateral, non-affected hemisphere (Michalski et al., 2017). This is in line with our data, which exhibit an increased microglial cell density in ischemia-affected brain regions. After an ischemic stroke, the blood-brain-barrier (BBB) is compromised (Latour et al., 2004; Sandoval and Witt, 2008; Krueger et al., 2015, 2017) and a BBB leakage coincides with an increased number of activated glial cells (Kuntz et al., 2014). Thus, a failing of the BBB integrity is followed by an infiltration of peripheral immune cells including neutrophils, lymphocytes, dendritic cells, and macrophages (microglia-derived and monocytes-derived macrophages) into the ischemic brain tissue (Kim and Cho, 2016; Jian et al., 2019). According to Rayasam et al. (2018), microglia in the CNS and peripheral immune cells are recruited to the ischemic hemisphere inducing an inflammatory response after stroke (Rayasam et al., 2018). Upon an ischemic event, microglial cells are the first responders and become activated within 30 min after cerebral ischemia (Rupalla et al., 1998), peak at 2–3 days post-stroke, and persist for several weeks (Denes et al., 2007; Gelderblom et al., 2009). Activated microglia and monocytes/macrophages are similar in morphology and function, but recent studies in rodent models of transient cerebral ischemia reported that microglia dominate the ischemic brain at day 1 and 2 after ischemia. For instance, on day 1, only a small fraction of monocytes/macrophages was determined (<3%; Jian et al., 2019; Rajan et al., 2019; Han et al., 2020). Thus, the here detected higher microglial cell density within ischemia-affected brain regions after 1 day of transient ischemia is predominantly provoked by resident microglial cells of the activated and rod-like morphotypes. However, at this point, the given data cannot provide any conclusion on the temporal evolution of microglia phenotypes post-stroke and the time course of the alterations will have to be investigated (Mages et al., 2021). Furthermore, it cannot be ruled out that activated Iba1-positive cells might also include monocytes/macrophages.

Our findings show that the segmented image area did not reveal any differences within the neocortical and hippocampal regions. This parameter reflects the proportion of segmented pixels—or the raw count of all stained structures—within the image area before cell detection. The values are comparable since the tissue volume is evenly permeated by cells and their processes as the task of microglial cells is to evenly monitor the tissue. But their number and distribution depend on the activation state of the microglial cells. In slides with high cell density, the cell territories are smaller. There we found a larger proportion of somata with connected processes as well as fewer processes belonging to somata located outside the imaged tissue slice. These images are characterized by a larger share of activated and rod-like cells with comparatively larger somata and shorter processes. In slides with low cell density, the cell territories are larger. There we found a smaller proportion of somata with connected processes as well as more processes belonging to somata located outside the imaged tissue slice. These images are characterized by a larger share of ramified cells with comparatively smaller somata and longer processes.

For verification of our classification method of microglial morphological phenotypes, we analyzed several morphological parameters of about 16,000 Iba1-positive cells in accordance with recently published studies (Kongsui et al., 2014; Zanier et al., 2015; Fernández-Arjona et al., 2017, 2019). Comparing studies of Zanier et al. (2015) and Fernández-Arjona et al. (2017, 2019) with our work showed that the *cell area* of our classified group of activated microglial cells is highly distributed in different brain regions and is not bigger than ramified *cell’s area* on average (Zanier et al., 2015; Fernández-Arjona et al., 2017, 2019). Here, we also examined rod-like microglial cells showing bigger *cell areas*. Activated and rod-like microglia in sum have bigger *cell areas* than ramified cells. In line with all studies including analysis of morphological parameters for microglial cells, we confirmed for instance larger *cell perimeters* and *convex hull areas* as well as smaller *cell soma areas* for ramified microglia.

Microglial cells are sensitive to fluctuations in blood flow and its reduction leads to a significant decrease in process activity and results in noticeable deramification and increased cell soma size (Masuda et al., 2011). Indeed, after ischemia, microglia tend to retract their fine, highly ramified processes leading to a reduced *skeleton length* what we have shown for all microglial cells within the ischemic neocortex and hippocampus as well as for activated compared to ramified cells. Reduced *branching indices* and *SRI* in ischemic compared to control regions confirmed this issue.

In contrast to Zanier et al. (2015), we distinguish between four different morphological classes of Iba1-stained cells in addition to an observation of all microglial cells after ischemia in control and ischemic-affected brain regions. The authors showed higher measurements for *cell area* and *cell perimeter* of CD11b-positive cells after transient MCA occlusion compared to naive mice. In line, we also used 20 μm thick brain slices for the analysis of microglial morphology (Zanier et al., 2015).

It has been recently shown by Fernández-Arjona et al. (2019) that, after injection of the enzyme neuraminidase within the lateral ventricle, activated microglial cells within the hypothalamus can be clustered in four different morphotypes characterized by various morphological parameters and IL-1β expression levels. Here, we were unable to cluster activated microglia due to strong overlapping between different value ranges of morphological parameters, which can be an argument for morphological classification and against pure quantification. Moreover, the authors analyzed 150 activated cells, whereas we examined thousands of Iba1-positive cells. Clustering with fewer microglial cells showing extreme morphological characteristics of the activated morphotype is more efficient than with numerous microglia classified by their probability. We should also take into account the heterogeneity of microglial cell density and morphology across different brain regions. Microglial cell morphology is affected by the cellular architecture of specific brain areas. Fernández-Arjona et al. (2017) suggested the consideration of the brain location for future microglial morphological classification (Fernández-Arjona et al., 2017, 2019).

While the classification of microglial cells solely based on parameters from quantitative analysis has been proven to be a successful approach (Kongsui et al., 2014; Zanier et al., 2015; Fernández-Arjona et al., 2017, 2019; Morrison et al., 2017; York et al., 2018; Kyriazis, 2019), our results show that parameter values may differ considerably within individual classes. There partially is a wide spread in parameter values caused by the biological variance and it needs to be considered that the highest probability is pivotal for the final morphological classification of microglial cells.

Small morphological differences can indicate an incipient change in the activation state of microglial cells. Such changes are detectable by a CNN and could give an indication of pathological processes in the brain. While our CNN covers four morphological states, it is not yet known if different activation states are physiologically relevant. Furthermore, transitions between different phenotypes are fluent and subtle. While classification based on four discrete classes provides a good distinction of these phenotypes, there is some latitude with regard to the morphology within these individual classes. There are differences in parameter expressions between different brain regions (activated microglia in the cortex differ from activated microglia in the hippocampus). Application of continuous scoring models like embedding visualization such as t-Distributed Stochastic Neighbor Embedding could be much more sensitive to even smaller morphological changes which should be explored in further studies.

CNN-based cell classification offers a powerful and interesting alternative to parameter-based cell classification. There are a number of morphological and topological parameters that are widely used to characterize microglial cells—some of them are basic properties (*cell area, skeleton length*, etc.), while others are combinations of multiple parameters (*circularity, branching index*, etc.). While we have presented 18 parameters in our study, there are many more that can be computed—and some parameters might be more significant for classification in a specific context than others.

Although the focus of this study is CNN-based cell classification, we also applied NC classification—a conventional parameter-based approach. While CNN classification is purely based on the shape of the cells, NC classification requires a set of morphological parameters that have to be computed prior to classification. As we have shown, it is not easy to determine a parameter set that is best suited for this task, since stronger parameter correlations may result in lower degrees of classification conformity. Without a thorough examination of all parameters for their interrelationships, it is not possible to make accurate predictions regarding their discriminatory power—but this would reach far beyond the scope of this study. While we have found potential parameter combinations and could compile a superficial ranking at best, the resulting classification quality is still inferior to results from CNN classification. Since NC classification is based on predefined centroids and class membership is assigned due to minimum centroid distances, different cluster shapes or sizes are not taken into account. Furthermore, this approach cannot generate overlapping classes by design, which—considering the high interpenetration of class point clusters—is a major strength of CNN classification. The NC approach also classifies a larger proportion of activated cells as rod-like cells, indicating a lower discriminatory power between these two classes. Due to the broad distribution of amoeboid cell parameters (*cell circularity* and *soma circularity*) or the strong overlap with clusters of the other three classes (*cell area* and *skeleton length*), amoeboid cells consistently show the highest number of NC misclassification in terms of their relative count. This is especially precarious regarding the low number of amoeboid cells within the images of our study.

Since it might be difficult to find suitable parameter combinations and specify thresholds for the assignment of cells to classes, we advocate for cell classification based on cell phenotype followed by quantitative analysis for morphological characterization. Cell classification based on CNNs does not require any parameters or combinations thereof, it is solely based on the cell’s shape represented as an image matrix. Deep learning-based approaches are becoming more accessible to researchers due to rapid technical progress and training CNNs on current graphics hardware with powerful GPUs gets increasingly time- and cost-efficient.

For the calculation of morphological parameters, a fully automatic approach was implemented and adapted to the characteristics of segmented cells. Manual analyses might be slightly better suited in cases of heterogeneous image quality or during interactive detection and reconstruction of interrupted cell processes, but they also greatly depend on the experience and endurance of the investigator. While automatic approaches require extensive testing and might introduce systematic errors, they are much faster than manual analyses and provide objective repeatability.

The proposed classification approach can be also applied to other staining and image acquisition setups as long as four key criteria are met: (1) staining quality and contrast must be sufficiently good to ensure reliable cell segmentation; (2) image resolution must be high enough to allow separation of cell processes during segmentation procedure; (3) tissue thickness must be chosen adequately to acquire (a) enough volume with a sufficient number of microglia showing an adequate amount of processes, while (b) avoiding overpopulated volumes with excessively interconnected network of ambiguously assignable cell processes; and (4) all segmented cells have to be adapted to match the input criteria for the CNN if an already trained CNN exists.

In the next step, we want to analyze microglia in scanned serial sections for 3D reconstruction and additionally in human brain tissue. Moreover, the morphological classification using machine learning can be transferred to other cell types like astrocytes and neurons.

In conclusion, our newly established classification method of microglial morphological phenotypes using machine learning represents an objective, unbiased and time-saving procedure that can serve as a powerful tool for post-mortem characterization of microglial changes in disease mouse models, and probably human brain autopsy samples.

## Data Availability Statement

The raw data supporting the conclusions of this article will be made available by the authors, without undue reservation.

## Ethics Statement

The animal study was reviewed and approved by the local ethics committee of the state of Saxony (Landesdirektion Sachsen, Leipzig, approval nos. TVV 65/15, TVV 02/17, and TVV 41/17).

## Author Contributions

JL, SP, IB, and KW conceived and designed the study. KW performed image acquisition and image processing. DM and BM carried out the animal experiments of cerebral ischemia. JL carried out the animal experiments of male wild-type C57BL/6J mice. MN provided and SP carried out the animal experiments of *db/db* and *db/+* mice. JL, SP, and KW analyzed the data and wrote the manuscript. All authors contributed to the article and approved the submitted version.

## Conflict of Interest

The authors declare that the research was conducted in the absence of any commercial or financial relationships that could be construed as a potential conflict of interest.

## Footnotes

^1^http://icy.bioimageanalysis.org

^2^http://www.R-project.org

## Abbreviations

CNS: central nervous system
CNN: convolutional neural network
DAPI: 4′,6-diamidino-2-phenylindole
PFA: paraformaldehyde
PBS: phosphate buffered saline
SRI: Schoenen ramification index
BBB: blood-brain barrier
DAMP: damage-associated molecular patterns
NF-L: neurofilament light
MAP2: microtubule-associated-protein-2
Coll IV: collagen IV
Iba1: ionized calcium-binding adapter molecule 1
ROI: Regions of interest
CLAHE: contrast limited adaptive histogram equalization
MCA: middle cerebral artery.
