# Supplementary material for: Classification of Microglial Morphological Phenotypes Using Machine Learning
Source: Front Cell Neurosci. 2021 Jun 29;15:701673. doi: 10.3389/fncel.2021.701673 (PMC8276040; doi:10.3389/fncel.2021.701673)
Supplement: Supplementary file 1 [file Data_Sheet_1.pdf]

Classification of microglial morphological phenotypes using machine learning

Supplementary Materials

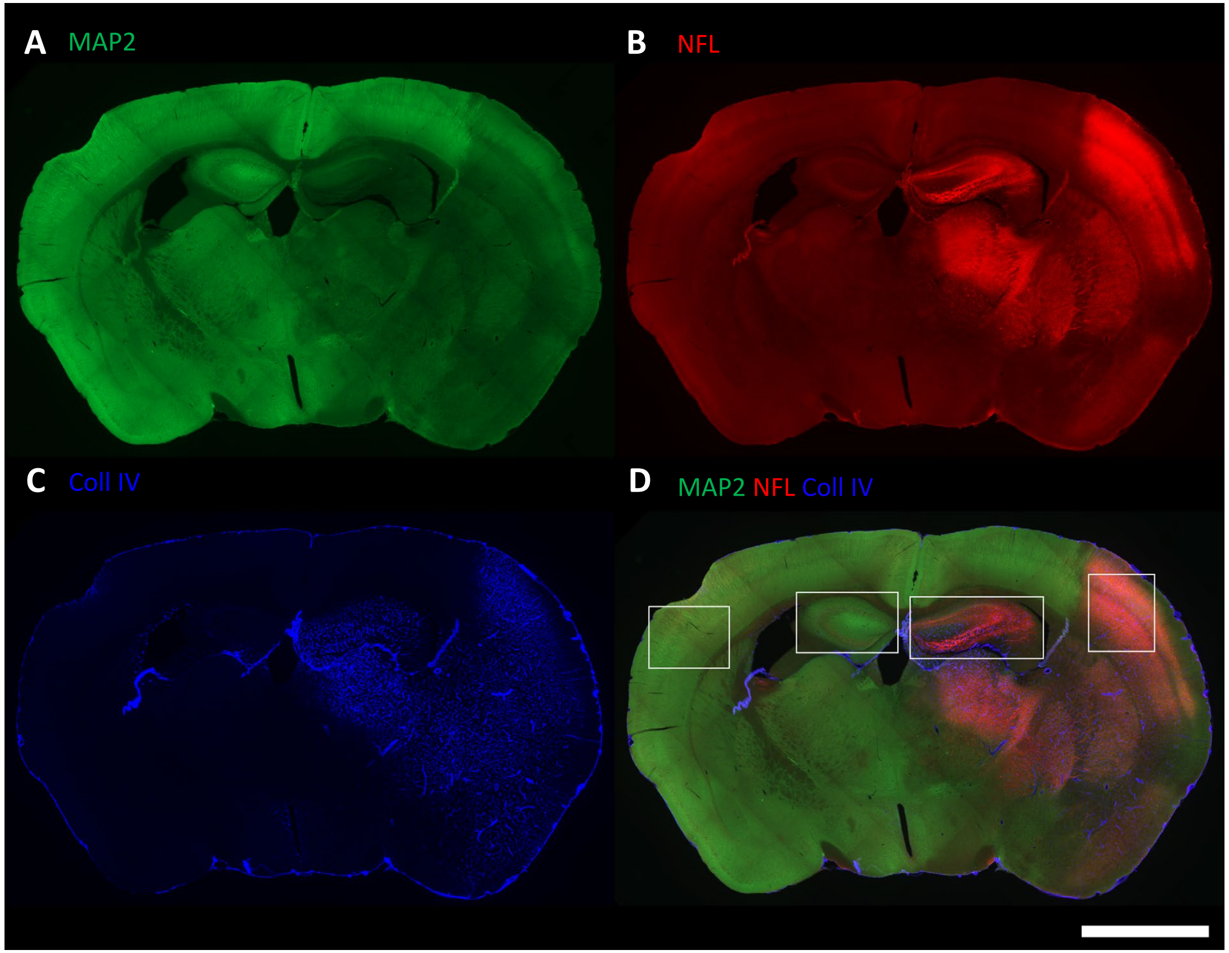

Supplementary Figure 1

Representative overview images of immunofluorescence staining for MAP2 (A), NFL (B), Coll IV (C), and triple immunofluorescence labelling of all applied markers (D) in mice 24 h after induction of experimental cerebral ischemia. Selected regions of interest in the hippocampus and neocortex within the ipsilateral, ischemia-affected hemisphere on the right and contralateral, control hemisphere on the left (F). Scale bar represents 200 µm.

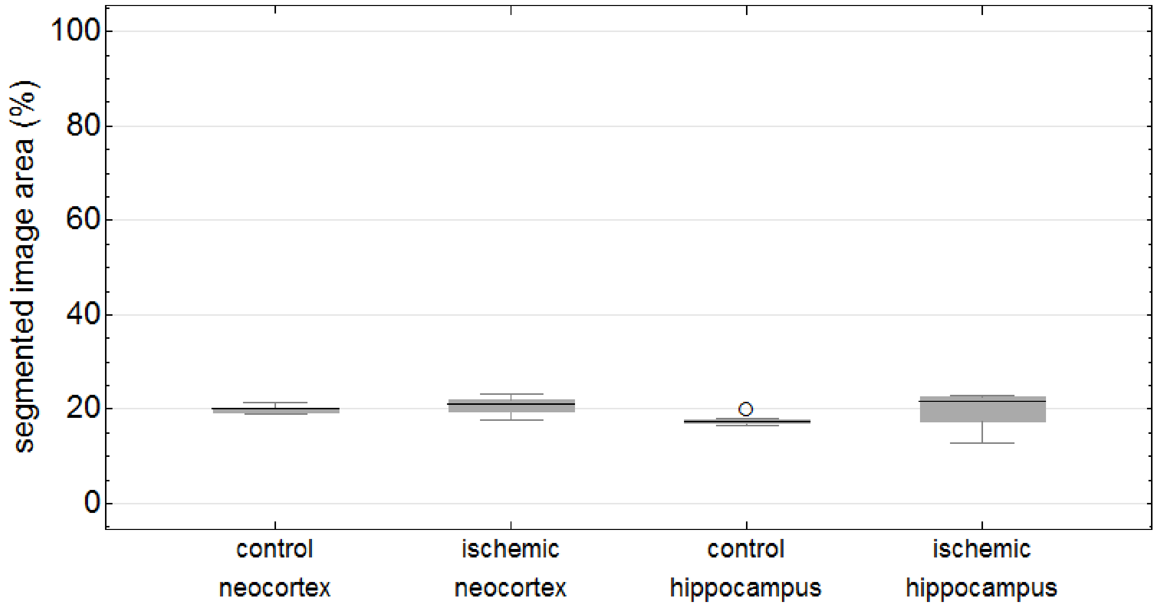

Supplementary Figure 2

Percentage of segmented image area in control and ischemic neocortex and in control and ischemic hippocampus.

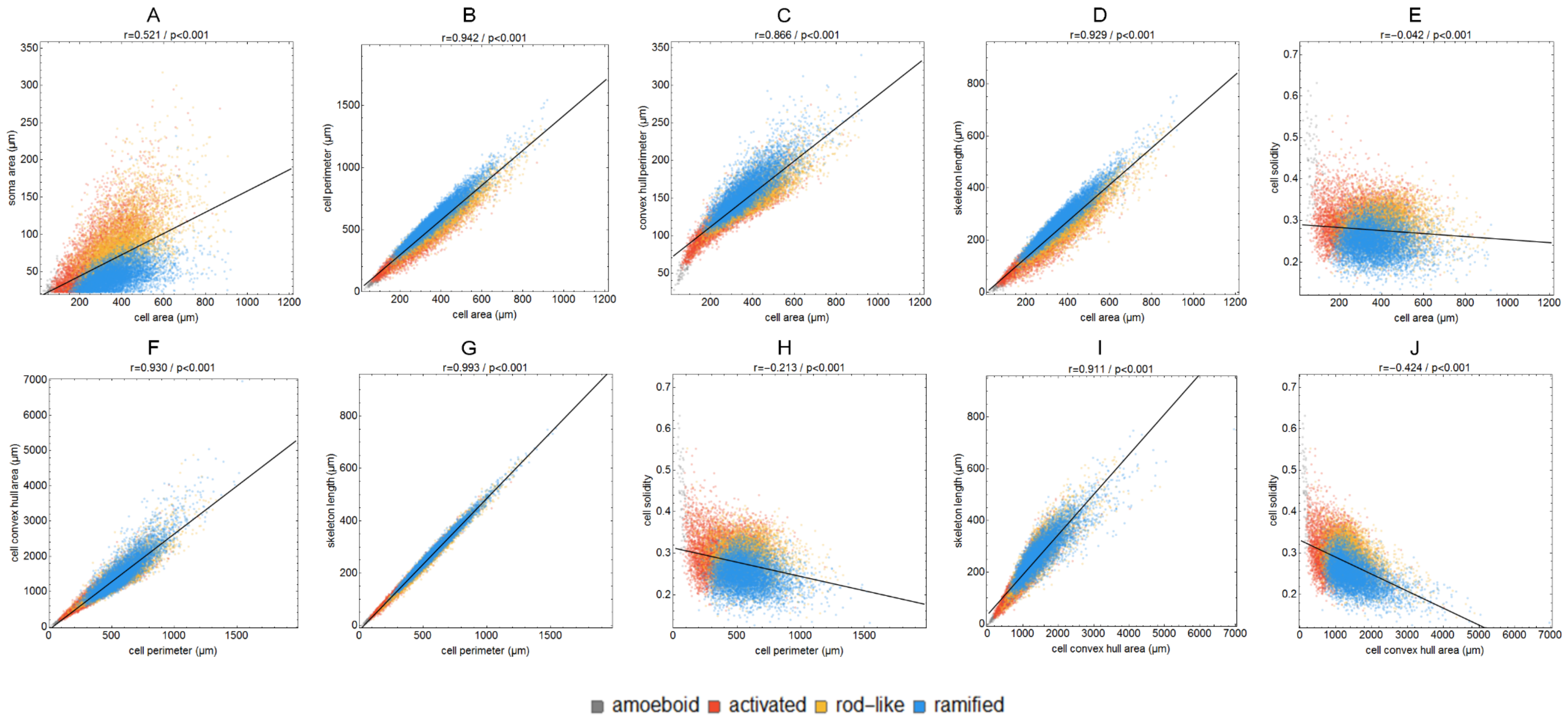

Supplementary Figure 3

Linear regression analysis between different microglial morphological parameters.

Supplementary Table 1: Morphological parameters of all microglial cells within control and ischemic neocortex and hippocampus.

parameter	control neocortex	ischemic neocortex	control hippocampus	ischemic hippocampus
**cells**	2024	4532	3105	6125
**cell area [µm²]**	375.10 (123.58)	374.85 (179.01)	317.12 (122.22)	320.01 (192.19)
**cell perimeter [µm]**	611.19 (203.45)	527.89 (244.06)	497.69 (193.26)	436.27 (262.61)
**convex hull area [µm²]**	1519.46 (655.09)	1263.45 (569.75)	1256.53 (588.97)	1110.21 (705.99)
**convex hull perimeter [µm]**	158.85 (37.14)	142.16 (34.81)	144.41 (35.58)	135.65 (43.06)
**cell solidity**	0.25 (0.04)	0.29 (0.05)	0.25 (0.05)	0.29 (0.06)
**cell convexity**	0.26 (0.05)	0.27 (0.08)	0.29 (0.07)	0.31 (0.11)
**cell circularity**	0.11 (0.02)	0.13 (0.03)	0.13 (0.03)	0.14 (0.05)
**soma area [µm²]**	43.04 (13.83)	74.23 (49.85)	43.15 (21.13)	66.26 (53.10)
**soma perimeter [µm]**	25.09 (4.74)	35.27 (14.50)	25.70 (7.56)	33.59 (17.08)
**soma circularity**	0.93 (0.04)	0.88 (0.09)	0.91 (0.06)	0.87 (0.11)
**skeleton length [µm]**	290.63 (100.90)	247.78 (126.40)	231.87 (97.68)	200.57 (132.59)
**skeleton branch points**	28 (12)	25 (17)	21 (12)	18 (15)
**skeleton end points**	18 (7)	16 (7)	15 (7)	14 (9)
**cell processes**	3 (2)	4 (2)	3 (2)	4 (3)
**branching index**	224.62 (137.11)	189.61 (126.41)	176.97 (122.52)	160.44 (136.13)
**critical radius**	12.64 (2.92)	12.64 (3.89)	12.64 (2.92)	11.67 (4.86)
**dendritic maximum**	14 (4)	14 (5)	12 (5)	11 (5)
**SRI**	5 (4.10)	3.25 (3.08)	3.67 (3.5)	2.50 (2.08)

Values are expressed as median (and interquartile range).

Supplementary Table 2: Morphological parameters of classified microglial cells.

parameter	amoeboid	activated	rod-like	ramified
**cells**	80	4337	5104	6265
**cell area [µm²]**	73.13 (33.92)	253.34 (189.69)	380.86 (166.35)	354.29 (137.28)
**cell perimeter [µm]**	76.12 (35.58)	333.22 (221.07)	533.51 (225.63)	557.59 (208.78)
**convex hull area [µm²]**	172.62 (94.97)	842.47 (554.43)	1316.72 (565.99)	1420.09 (641.69)
**convex hull perimeter [µm]**	56.36 (17.12)	118.43 (36.28)	146.42 (33.65)	154.09 (36.48)
**cell solidity**	0.43 (0.12)	0.30 (0.06)	0.29 (0.05)	0.25 (0.05)
**cell convexity**	0.76 (0.19)	0.35 (0.13)	0.28 (0.07)	0.28 (0.06)
**cell circularity**	0.39 (0.11)	0.17 (0.05)	0.13 (0.03)	0.12 (0.02)
**soma area [µm²]**	28.93 (13.00)	68.33 (61.50)	79.23 (41.88)	42.03 (18.9)
**soma perimeter [µm]**	20.59 (5.09)	33.02 (16.89)	38.65 (14.11)	25.01 (6.30)
**soma circularity**	0.93 (0.06)	0.89 (0.07)	0.82 (0.11)	0.92 (0.04)
**skeleton length [µm]**	24.59 (17.42)	149.47 (108.93)	247.72 (114.58)	264.81 (105.22)
**skeleton branch points**	1 (2)	13 (13)	24 (15)	25 (13)
**skeleton end points**	4 (2)	12 (8)	17 (8)	17 (7)
**cell processes**	3 (2)	4 (3)	4 (3)	3 (2)
**branching index**	17.5 (12.64)	116.69 (99.18)	197.39 (125.44)	211.01 (141.97)
**critical radius**	3.89 (1.94)	10.70 (4.86)	13.61 (2.92)	12.64 (2.92)
**dendritic maximum**	3 (1)	10 (6)	14 (5)	13 (4)
**SRI**	1 (0.58)	2.20 (1.75)	3 (2.67)	4.33 (4.00)

Values are expressed as median (and interquartile range).

Supplementary Table 3: Morphological parameters of classified microglial cells within control and ischemic neocortex and hippocampus.

	amoeboid				activated				rod-like				ramified			
parameter	control neocortex	ischemic neocortex	control hippocampus	ischemic hippocampus	control neocortex	ischemic neocortex	control hippocampus	ischemic hippocampus	control neocortex	ischemic neocortex	control hippocampus	ischemic hippocampus	control neocortex	ischemic neocortex	control hippocampus	ischemic hippocampus
cells	0	5	2	73	55	1541	415	2326	209	1872	747	2276	1760	1114	1941	1450
cell area [µm²]	-	91.95 (40.32)	62.73 (8.57)	73.60 (31.26)	206.39 (66.33)	332.38 (219.45)	190.80 (91.64)	236.95 (159.95)	362.53 (142.42)	410.82 (162.19)	334.68 (109.68)	381.55 (171.74)	379.82 (120.67)	359.95 (153.71)	330.53 (114.26)	352.65 (175.65)
cell perimeter [µm]	-	93.06 (62.13)	62.21 (19.02)	76.35 (33.49)	312.16 (105.19)	429.68 (276.34)	289.35 (125.85)	306.75 (179.13)	558.33 (202.67)	574.82 (218.76)	503.10 (169.70)	509.60 (228.39)	621.36 (190.82)	545.45 (208.12)	527.82 (178.84)	526.71 (240.61)
convex hull area [µm²]	-	222.12 (137.29)	147.71 (73.44)	172.17 (79.08)	741.59 (298.03)	1049.51 (626.64)	722.23 (316.63)	780.12 (495.57)	1365.52 (632.64)	1346.30 (507.98)	1241.22 (514.66)	1303.97 (631.18)	1556.47 (642.20)	1339.29 (644.55)	1360.11 (573.62)	1360.77 (746.93)
convex hull perimeter [µm]	-	67.65 (32.07)	50.05 (10.91)	56.54 (15.99)	109.84 (15.88)	129.17 (38.07)	108.83 (23.07)	114.58 (34.50)	149.15 (38.61)	147.20 (29.99)	142.89 (29.95)	146.58 (37.23)	161.32 (36.19)	149.59 (36.14)	151.19 (34.12)	152.47 (41.81)
cell solidity	-	0.43 (0.13)	0.44 (0.16)	0.43 (0.12)	0.28 (0.04)	0.31 (0.06)	0.28 (0.05)	0.31 (0.06)	0.27 (0.06)	0.30 (0.05)	0.26 (0.04)	0.29 (0.05)	0.24 (0.04)	0.26 (0.05	0.24 (0.04)	0.25 (0.05)
cell convexity	-	0.73 (0.10)	0.81 (0.07)	0.76 (0.18)	0.36 (0.09)	0.30 (0.11)	0.38 (0.11)	0.38 (0.13)	0.27 (0.05)	0.26 (0.06)	0.29 (0.06)	0.029 (0.07)	0.26 (0.04)	0.27 (0.06)	0.29 (0.06)	0.29 (0.07)
cell circularity	-	0.37 (0.18)	0.46 (0.11)	0.38 (0.11)	0.16 (0.03)	0.15 (0.04)	0.17 (0.04)	0.18 (0.05)	0.12 (0.02)	0.12 (0.02)	0.13 (0.02)	0.13 (0.03	0.11 (0.02)	0.12 (0.02)	0.12 (0.02)	0.13 (0.03)
soma area [µm²]	-	30.49 (2.7)	38.01 (3.74)	28.60 (13.28)	43.04 (19.53)	86.28 (56.92)	40.91 (22.34)	66.64 (60.34)	56.94 (19.38)	85.16 (38.31)	60.07 (25.01)	84.45 (46.14)	41.83 (12.73)	48.54 (29.05)	39.87 (15.87)	44.15 (27.94)
soma perimeter [µm]	-	20.96 (1.33)	24.55 (1.50)	20.57 (5.54)	25.34 (5.70)	36.95 (14.77)	25.05 (7.39)	32.71 (17.02)	31.73 (6.97)	39.40 (12.37)	33.04 (9.99)	41.07 (16.39)	24.68 (4.34)	27.14 (8.52)	24.36 (5.55)	25.94 (9.02)
soma circularity	-	0.93 (0.02)	0.89 (0.01)	0.93 (0.06)	0.93 (0.04)	0.89 (0.06)	0.92 (0.05)	0.88 (0.08)	0.85 (0.07)	0.83 (0.10)	0.83 (0.09)	0.80 (0.12)	0.93 (0.03)	0.92 (0.04)	0.93 (0.04)	0.92 (0.05)
skeleton length [µm]	-	34.57 (33.98)	14.17 (10.24)	24.64 (16.58)	134.94 (45.02)	197.00 (139.42)	128.61 (62.31)	136.15 (91.29)	266.44 (98.87)	270.73 (114.77)	232.24 (85.02)	234.65 (113.19)	295.38 (98.38)	261.21 (108.84)	247.72 (87.95)	251.37 (120.50)
skeleton branch points	-	2 (4)	0	1 (2)	11 (7)	18 (17)	10 (7)	11 (11)	26 (13)	28 (17)	21 (11)	21 (14)	28 (11)	26 (14)	23 (11)	24 (14)
skeleton end points	-	4 (3)	4 (2)	4 (2)	10 (4)	14 (8)	10 (4)	11 (6)	17 (6)	18 (7)	16 (6)	17 (8)	18 (7)	17 (7)	16 (7)	17 (8)
cell processes	-	4 (1)	4 (2)	3 (2)	4 (2)	4 (3)	4 (2)	5 (3)	3 (3)	4 (3)	4 (2)	5 (2)	3 (2)	3 (2)	3 (2)	3 (2)
branching index	-	16.53 (10.70)	14.59 (1.94)	18.48 (12.64)	102.10 (48.62)	151.68 (115.71)	90.43 (56.4)	105.99 (141.97)	189.61 (130.30)	211.98 (124.46)	176.00 (102.10)	195.45 (128.35)	231.43 (134.19)	207.12 (140.029	198.36 (128.35)	207.12 (159.47)
critical radius	-	3.89 (0)	4.86 (0)	3.89 (1.94)	9.72 (3.89)	11.67 (4.86)	9.72 (5.83)	9.72 (4.86)	13.61 (2.92)	13.61 (2.92)	12.64 (3.89)	13.61 (4.86)	12.64 (2.92)	12.64 (2.92)	12.64 (2.92)	12.64 (3.89)
dendritic maximum	-	4 (1)	3.5 (1)	3 (1)	10 (5)	12 (6)	9 (4)	9 (5)	14 (3)	15 (5)	13 (4)	13 (5)	14 (4)	14 (5)	13 (4)	12 (4)
SRI	-	0.83 (0.25)	0.90 (0.20)	1 (0.53)	2.67 (2.00)	2.60 (2.17)	2.20 (1.50)	2 (1.40)	4.00 (3.75)	3.40 (3.08)	3.25 (2.71)	2.50 (1.96)	5.00 (4.50)	4.33 (3.87)	4.33 (4.00)	3.50 (3.00)

Values are expressed as median (and interquartile range).

Supplementary Table 4: Morphological parameters of classified microglial cells within control and ischemic neocortex and hippocampus.

		cell area [µm²]	cell perimeter [µm]	convex hull area [µm²]	convex hull perimeter [µm]	cell solidity	cell convexity	cell circularity	soma area [µm²]	soma perimeter [µm]	soma circularity	skeleton length [µm]	skeleton branch points	skeleton end points	cell processes	branching index	critical radius	dendritic maximum	SRI
amoeboid	iCx vs cHc	ns	ns	ns	ns	ns	ns	ns	ns	ns	ns	ns	ns	ns	ns	ns	ns	ns	ns
	iCx vs iHc	ns	ns	ns	ns	ns	ns	ns	ns	ns	ns	ns	ns	ns	ns	ns	ns	ns	ns
	cHc vs iHc	ns	ns	ns	ns	ns	ns	ns	ns	ns	ns	ns	ns	ns	ns	ns	ns	ns	ns
activated	cCx vs iCx	****	****	****	****	****	****	****	****	****	****	****	****	****	****	****	****	****	ns
	cCx vs cHc	ns	ns	ns	ns	ns	ns	ns	ns	ns	ns	ns	ns	ns	ns	ns	ns	ns	ns
	cCx vs iHc	ns	ns	ns	ns	****	ns	****	****	****	****	ns	ns	ns	****	ns	ns	ns	****
	iCx vs cHc	****	****	****	****	****	****	****	****	****	****	****	****	****	****	****	****	****	****
	iCx vs iHc	****	****	****	****	ns	****	****	****	****	ns	****	****	****	ns	****	****	****	****
	cHc vs iHc	****	****	****	****	****	****	ns	****	****	****	****	****	****	****	****	****	****	****
rod-like	cCx vs iCx	****	ns	ns	ns	****	ns	****	****	****	****	ns	ns	ns	****	ns	ns	****	****
	cCx vs cHc	****	****	ns	****	ns	****	****	ns	ns	****	****	****	****	*	****	ns	****	****
	cCx vs iHc	ns	****	ns	ns	****	****	****	****	****	****	****	****	ns	****	ns	ns	****	****
	iCx vs cHc	****	****	*	****	****	****	****	****	****	****	****	****	****	****	****	****	****	ns
	iCx vs iHc	****	****	ns	****	****	****	****	ns	****	****	****	****	****	****	****	ns	****	****
	cHc vs iHc	****	ns	ns	ns	****	ns	****	****	****	****	ns	ns	****	****	****	****	ns	****
ramified	cCx vs iCx	****	****	****	****	****	****	****	****	****	****	****	****	****	****	****	ns	****	****
	cCx vs cHc	****	****	****	****	ns	****	****	****	***	****	****	****	****	**	****	****	****	****
	cCx vs iHc	****	****	****	****	****	****	****	****	****	****	****	****	****	****	****	****	****	****
	iCx vs cHc	****	****	ns	ns	****	****	ns	****	****	****	****	****	****	****	****	ns	****	ns
	iCx vs iHc	ns	****	*	ns	****	****	****	****	**	ns	ns	****	ns	*	ns	ns	****	****
	cHc vs iHc	****	ns	ns	ns	****	ns	****	****	****	****	ns	****	ns	****	****	ns	****	****

cNCX – control neocortex; iNCX- ischemic neocortex; cHC- control hippocampus; iHC- ischemic hippocampus; ns – not significant; $p < 0.05$ *, $p < 0.01$ **, $p < 0.001$ ***, $p < 0.0001$ ****
